# Scent test using *Caenorhabditis elegans* to screen for early-stage pancreatic cancer

**DOI:** 10.18632/oncotarget.28035

**Published:** 2021-08-17

**Authors:** Ayumu Asai, Masamitsu Konno, Miyuki Ozaki, Koichi Kawamoto, Ryota Chijimatsu, Nobuaki Kondo, Takaaki Hirotsu, Hideshi Ishii

**Affiliations:** ^1^Center of Medical Innovation and Translational Research (CoMIT), Graduate School of Medicine, Osaka University, Suita, Osaka, Japan; ^2^Artificial Intelligence Research Center, Institute of Scientific and Industrial Research, Osaka University, Ibaraki, Osaka, Japan; ^3^Hirotsu Bio Science Inc., Chiyoda-Ku, Tokyo 102-0094, Japan; ^4^Present address: Research Institute for Biomedical Sciences, Tokyo University of Science, Chiba, Japan; ^5^Present address: Kinnki Regional Bureau of Health and Welfare, Osaka, Japan

**Keywords:** *Caenorhabditis elegans*, early-stage pancreatic cancer, scent test, biomarker, diagnosis

## Abstract

Although early detection and diagnosis are indispensable for improving the prognosis of patients with pancreatic cancer, both have yet to be achieved. Except for pancreatic cancer, other cancers have already been screened through scent tests using animals or microorganisms, including *Caenorhabditis elegans*. While such a method may greatly improve the prognosis of pancreatic cancer, no studies have investigated the same, mainly given the difficulty of collecting suitable samples from patients with early-stage pancreatic cancer. In this study, we organized a nationwide study group comprising high-volume centers throughout Japan to collect patients with very-early-stage pancreatic cancer (stage 0 or IA). We initially performed an open-label study involving 83 cases (stage 0–IV), with subsequent results showing significant differences after surgical removal in stage 0–IA (×10 dilution: *p* < 0.001; ×100 dilution: *p* < 0.001). Thereafter, a blinded study on 28 cases (11 patients with stage 0 or IA disease and 17 healthy volunteers) was conducted by comparing very-early-stage pancreatic cancer patients with healthy volunteers to determine whether *C. elegans* could detect the scent of cancer for the diagnosis of early-stage pancreatic cancer. Preoperative urine samples had a significantly higher chemotaxis index compared to postoperative samples in patients with pancreatic cancer [×10 dilution: *p* < 0.001, area under the receiver operating characteristic curve (AUC) = 0.845; ×100 dilution: *p* < 0.001, AUC = 0.820] and healthy volunteers (×10 dilution: *p* = 0.034; ×100 dilution: *p* = 0.088). Moreover, using the changes in preoperative and postoperative chemotaxis index, this method had a higher sensitivity for detecting early pancreatic cancer compared to existing diagnostic markers. The clinical application *C. elegans* for the early diagnosis of cancer can certainly be expected in the near future.

## INTRODUCTION

Pancreatic ductal adenocarcinoma (PDAC) is among the deadliest diseases, with a five-year survival rate of 9% [1]. Although surgical resection is the only curative treatment, less than 10% of cases are surgically resectable, and the median survival is only 17–23 months even after successful resection [2]. On the other hand, studies have reported a 5-year survival rate of 80.4% for PDAC smaller than 10 mm (TS1a) and 85.8% for Union Internationale Contre le Cancer (UICC) stage 0, indicating that early detection of PDAC is indispensable for overcoming this refractory disease [3, 4]. Yachida et al. reported that the duration from the onset of pancreatic mutation to the development of metastatic PDAC takes almost 21 years, which consequently provides enough time for detection before progression into the advanced stage [5]. However, imaging modalities have remained the currently available practical methods for detecting PDAC, making it almost impossible to distinguish patients with very-early-stage (stage 0 or IA) PDAC.

Various studies involving genomics, transcriptomics, proteomics, and metabolomics have identified putative molecular biomarkers for the diagnosis of early-stage PDAC, some of which analyzed body fluids, such as feces, urine, and saliva, of patients with PDAC [6, 7]. However, most of these molecular biomarkers still remain unavailable or under development. On the other hand, several reports have emerged regarding canine scent detection of cancers. Since Williams’ first report regarding the ability of sniffer dogs to detect skin cancer, a number of studies have indicated the ability of dogs to detect cancerous lesions, including those in the bladder, lungs, breast, and ovaries [8–12]. However, Williams also reported that canine scent diagnosis may be difficult to introduce into clinical practice owing to the expenses and time required for canine training and education.

Similar to sniffer dogs, the use of *Caenorhabditis elegans* has been introduced as a new strategy for detecting cancer-associated scents during cancer screening. Aside from having an excellent sense of smell, *C. elegans* is easy to handle, inexpensive, and quick to breed. The method for olfaction analysis, named N-NOSE (Nematode-NOSE), has been well established as a simple system for observing chemotaxis. These features make this organism ideal for screening tests [13, 14]. Hirotsu et al. reported that wild-type *C. elegans* displayed attractive chemotaxis toward human cancer cell secretions, cancer tissues, and urine from patients with colorectal, gastric, and breast cancers [15]. This biological diagnosis had a reported sensitivity of 95.8%, which was also acceptable even in patients in early-stage of cancer. Furthermore, reports have shown that this test demonstrated high sensitivity in cases of gastrointestinal cancers and negative changes in the postoperative period [16, 17]. Moreover, this test could discriminate urine in a mouse model of pancreatic cancer [18]. Therefore, this method may be useful for detecting patients with early PDAC. However, how study has yet utilized this method to detect very-early-stage (stage 0 or IA) PDAC mainly due to the extreme difficulty of collecting urine samples from such patients [6]. In the present study, we organized a nationwide clinical group that comprised high-volume centers throughout Japan and prospectively collected serum and urine samples from patients with very-early-stage PDAC (stage 0 or IA) to investigate the clinical value of a cancer detection system involving *C. elegans*.

## RESULTS

**Figure 1:**
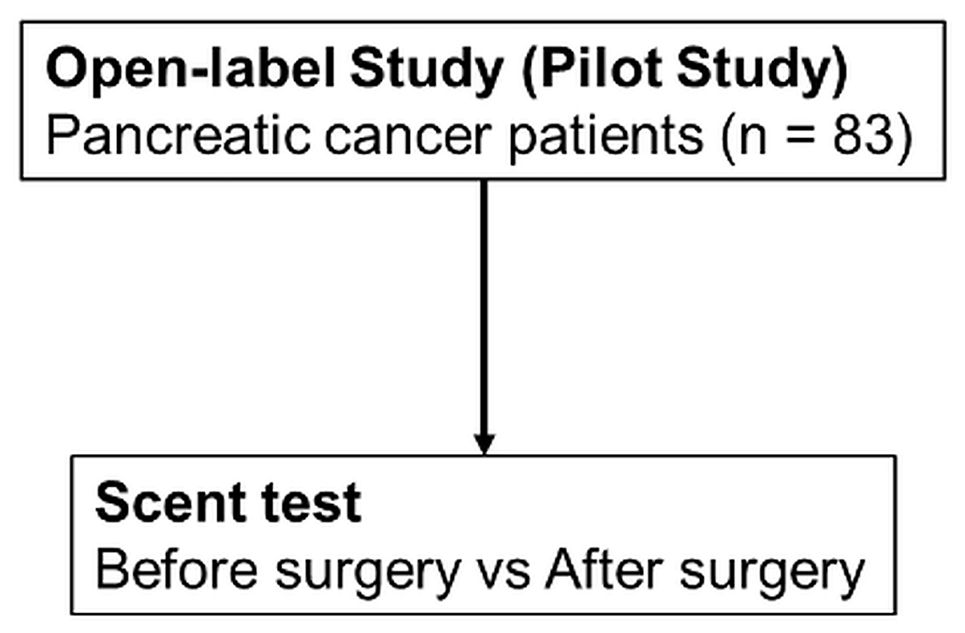
Schema of the scent test using *Caenorhabditis elegans* for the screening of pancreatic cancer in the open-label study.

**Figure 2:**
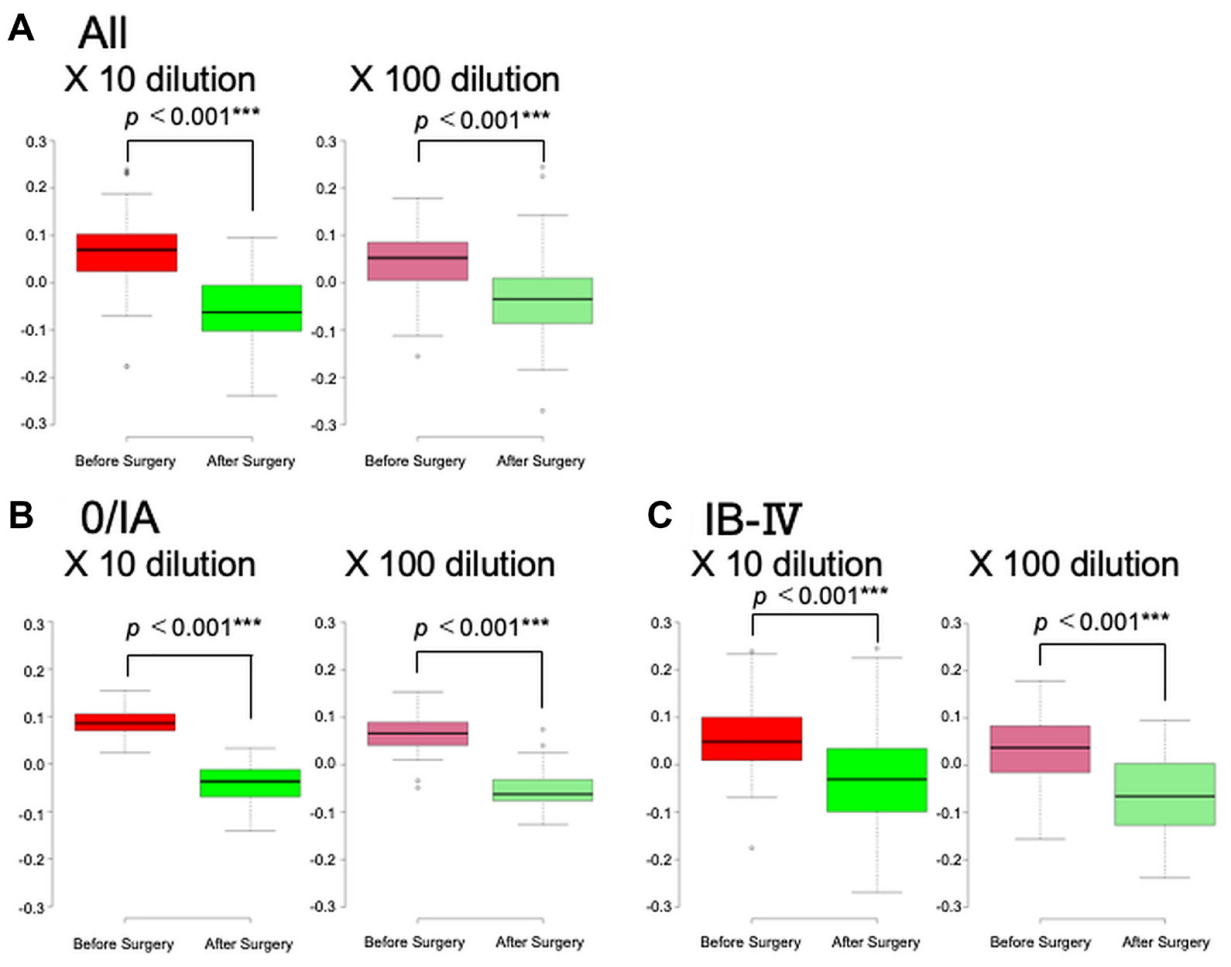
Scent test using *Caenorhabditis elegans* before and after surgical removal of pancreatic ductal adenocarcinoma. The chemotaxis index was measured in 83 cases. The scent tests were performed in cases of all pancreatic cancers (**A**), stages 0/IA (**B**), and IB–IV (**C**).

**Figure 3:**
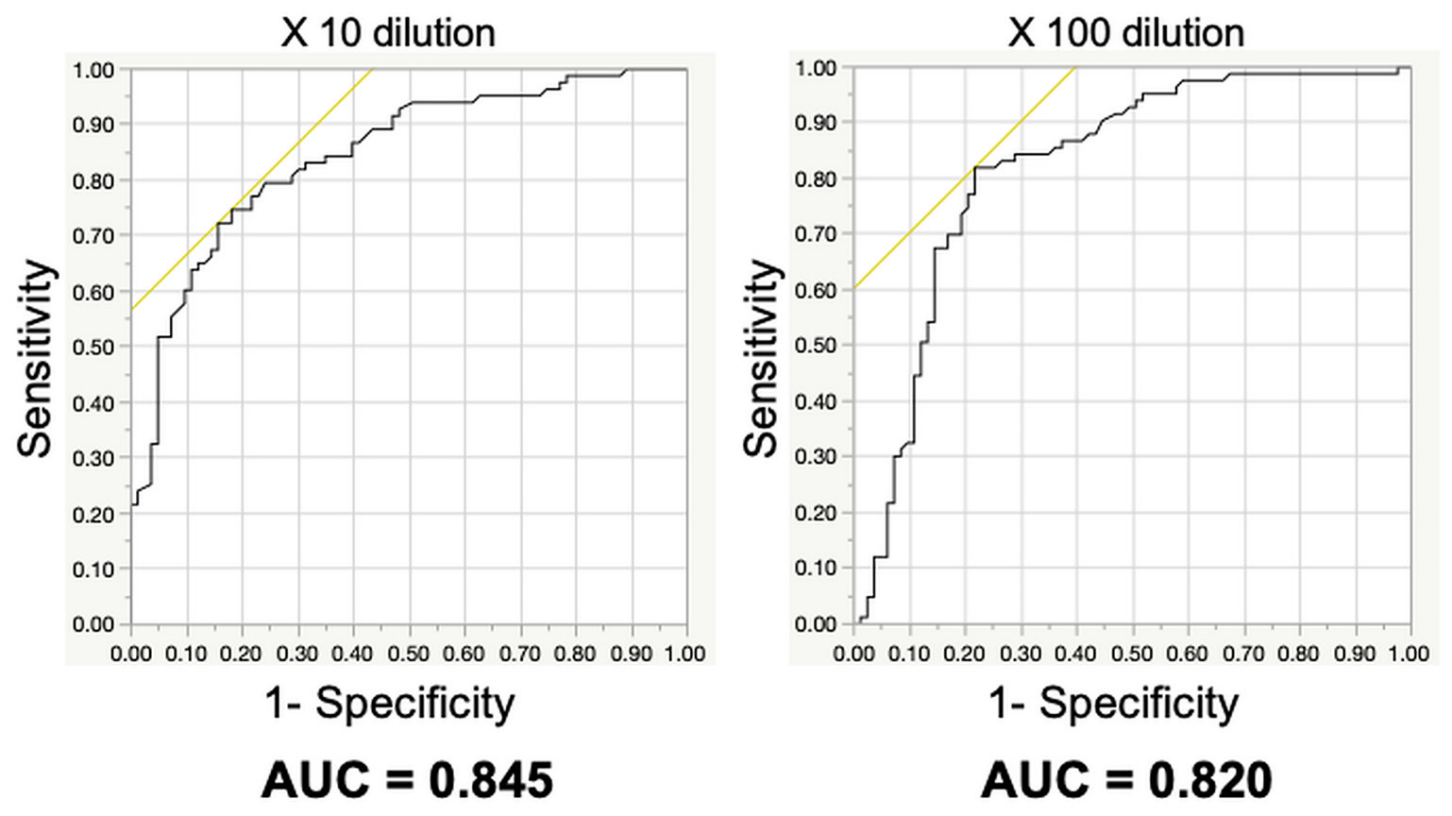
Diagnostic accuracy of the scent test using *Caenorhabditis elegans* before and after surgical removal of pancreatic ductal adenocarcinoma.

Prior to the study on early-stage pancreatic cancer, 83 patients with various stages of pancreatic cancer were investigated in an open-label pilot study (Figure 1). The age, sex, pathological progression, CA19-9 value, and CEA value for the 83 patients are summarized in Table 1. Results of the scent test using urine samples from the 83 patients with PDAC showed a significant decrease in the chemotaxis index of postoperative urine samples at both 10- and 100-fold dilutions (Figure 2A). To evaluate the effects of cancer progression on the scent test, early-stage (0, IA) and late-stage samples (IB–IV) were investigated. Accordingly, both early- and late-stage postoperative urine samples showed a decrease in the chemotaxis index (Figure 2B, 2C). Furthermore, to evaluate the diagnostic performance of the scent test for the presence of tumor tissue (pre-/postoperative), a receiver operating characteristic (ROC) curve was created. Subsequent results showed that the scent test had high performance with an area under the (ROC) curve (AUC) of 0.845 and 0.820 at 10- and 100-fold dilutions (Figure 3).

**Table 1:** Characteristics of patients enrolled in the open-label study

	PDAC
Number of cases	83
Age	71.0 ± 8.7 (41–87)
Sex (M/F)	56/27
Pathological stage (0/IA/IB/IIA/IIB/III/IV)	5/20/4/28/23/0/3
CA19-9	31.0 ± 1279.7 (0.6–8997.0)
CEA	2.7 ± 3.8 (0.3–29.0)

**Figure 4:**
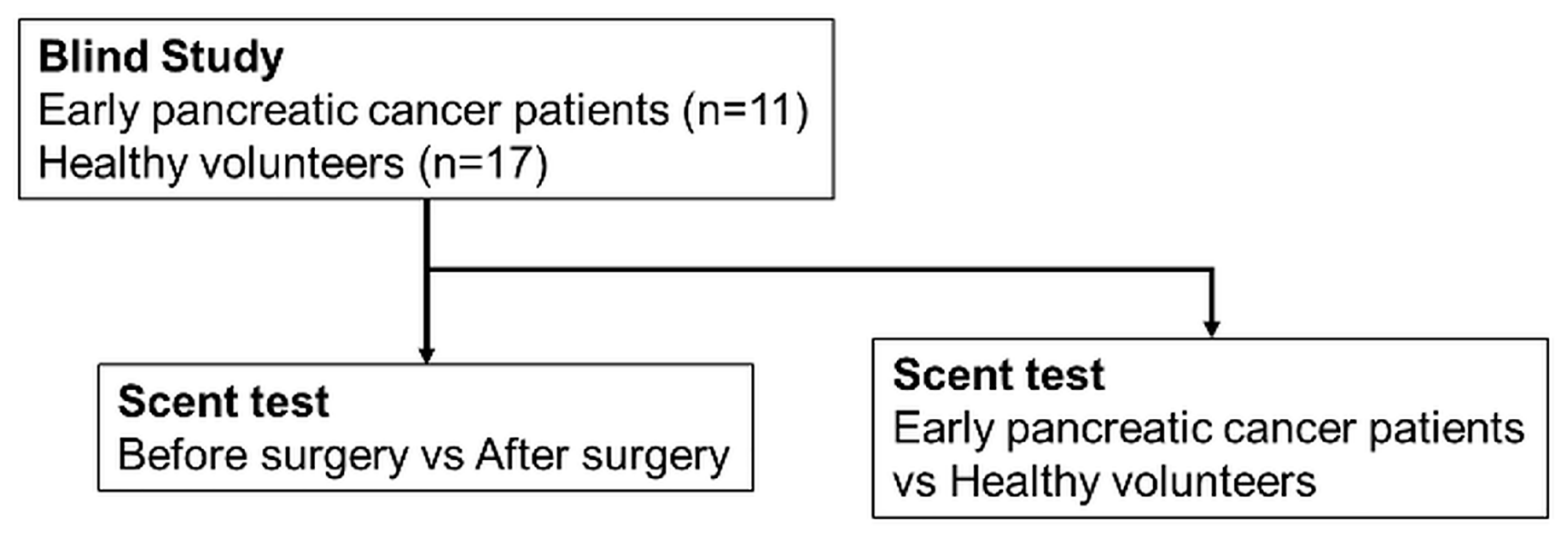
Schema of the scent test using *Caenorhabditis elegans* for the screening of pancreatic cancer in the blinded study.

Given the success of the scent test in determining the presence of pancreatic cancer in patients with PDAC who showed various progressions before and after surgery, a blinded study was conducted to determine the ability of the scent test to distinguish between patients with early-stage PDAC and healthy volunteers (Figure 4). The age, sex, pathological progression, CA19-9 value, and CEA value of 11 stage 0 or IA cases are detailed in Table 2. Given that all cases had early-stage pancreatic cancer, the tumor marker values were normal in most cases. Among the 17 healthy volunteers, 1 had slightly elevated CA19-9, whereas all the other subjects had normal CA19-9 and CEA.

**Table 2:** Characteristics of patients enrolled in the blinded study

	Early PDAC	Healthy Volunteers
Number of cases	11	17
Age	70.6 ± 7.8 (58–84)	39.8 ± 8.3 (30–55)
Sex	4/7	17/0
Pathological stage (0/IA)	3/8	(–)
CA19-9	23.2 ± 18.9 (2–58)	12.7 ± 10.6 (1–41.3)
CEA	3.0 ± 1.3 (0.8–4.8)	2.0 ± 1.1 (0.5–4.0)

**Figure 5:**
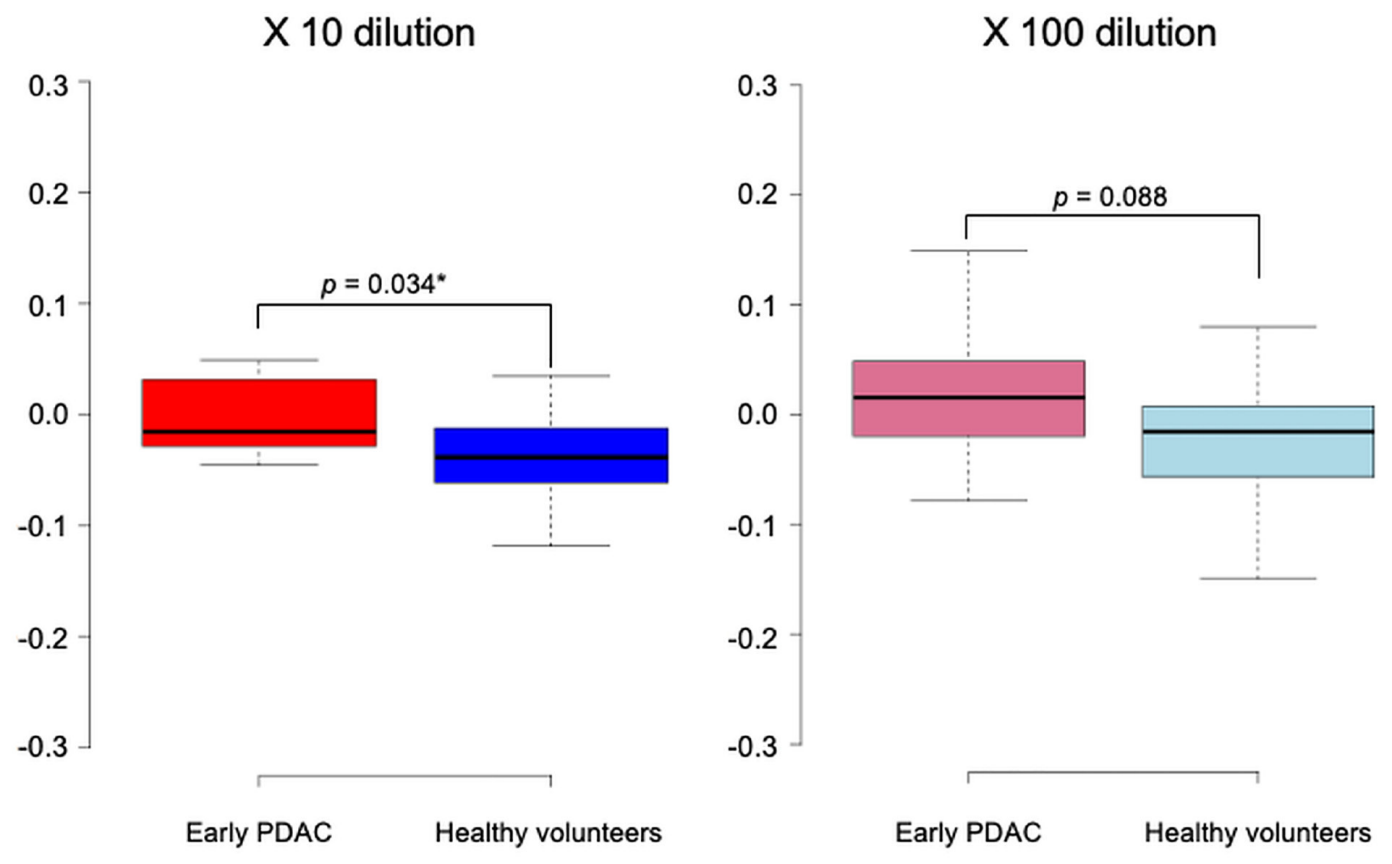
Scent test using *Caenorhabditis elegans* for the diagnosis of early pancreatic ductal adenocarcinoma. The chemotaxis index was measured in 11 early pancreatic ductal adenocarcinoma cases and 17 healthy volunteers.

The chemotaxis index was measured in 11 early PDAC cases and 17 healthy volunteers. Among the urine samples diluted 10-fold, early PDAC cases had a significantly higher median index compared to healthy volunteers [−0.015 (range, −0.045 to 0.049) vs. −0.038 (range, −0.118 to 0.036); *p* = 0.034] (Figure 5). When the dilution of the urine sample was further increased to 100-fold, the early PDAC cases tended to have a higher median index compared to healthy volunteers [0.016 (range, −0.078 to 0.149) vs. −0.015 (range, −0.149 to 0.080); *p* = 0.088].

On the other hand, the sensitivity of CEA and CA19-9 were 0% (0/11) and 27% (3/11) respectively, indicating the general difficulty of detecting early-stage pancreatic cancer (Table 3). In urine samples from early-stage PDAC cases in the blinded study, the chemotaxis index decreased after surgery, similar to that in the open-label study, although not significantly (Supplementary Figure 1). However, Table 3 shows that the chemotaxis index of approximately 50% of the patients decreased following surgery (10-fold dilution: 5/11; 100-fold dilution: 6/11), suggesting that the chemotaxis index could be a marker for the sensitive detection of early-stage pancreatic cancer.

**Table 3:** Patients with early-stage pancreatic cancer

Age	Sex	Tumor diameter (mm)	Tumor location	Pathology	ly	v	*p* Stage	CEA (ng/ml)	CA19-9 (U/ml)	Preoperative	Postoperative	Postoperative index − Preoperative index Δ index (×10)	Preoperative	Postoperative	Postoperative index − Preoperative index Δ index (×100)	Days after operation for postoperative sampling
										N-NOSE index (×10)	N-NOSE index (×10)		N-NOSE index (×100)	N-NOSE index (×100)		
74	F	4	Ph	Carcinoma *in situ*	0	0	0	4.8	16.3	0.044	−0.036	−0.080^*^	0.016	−0.015	−0.031^*^	33
67	F	8	Pb	Carcinoma *in situ*	0	0	0	0.8	2.5	−0.028	−0.051	−0.023^*^	0.004	0.003	−0.001^*^	85
84	F	13	Pb	Carcinoma *in situ*	0	0	0	1.4	7.8	0.020	−0.055	−0.075^*^	0.048	0.002	−0.046^*^	38
66	M	10	Ph	Poorly differentiated	0	0	IA	3.0	18.4	−0.015	−0.004	0.011	0.050	−0.039	−0.089^*^	42
82	F	18	Ph	Moderately differentiated	0	2	IA	3.6	50.8^*^	−0.021	−0.031	−0.010^*^	−0.038	−0.063	−0.025^*^	41
66	F	10	Ph	Moderately differentiated	0	0	IA	3.4	24.0	0.049	0.090	0.041	0.047	−0.021	−0.068^*^	27
76	M	16	Ph	Moderately differentiated	0	0	IA	4.7	58.0^*^	0.048	−0.095	−0.143^*^	−0.078	−0.073	0.005	84
69	F	18	Ph	Moderately differentiated	1	1	IA	1.7	18.9	−0.038	−0.035	0.003	0.001	0.059	0.058	41
71	M	10	Ph	Well differentiated	0	0	IA	3.8	15.0	−0.045	0.020	0.065	0.123	0.124	0.001	35
58	M	10	Pb	Well differentiated	0	0	IA	3.9	2.0	−0.028	0.009	0.037	−0.055	0.008	0.063	34
64	F	2	Ph	Well differentiated	0	0	IA	2.0	41.0^*^	0.003	0.093	0.090	0.149	0.237	0.088	84

Abbreviations: Ph: pancreatic head; Pb: pancreatic body; ly: lymph node invasion; venous invasion; pStage, pathological stage. According to the pancreatic cancer database of the Japanese Pancreas Society (http://www.suizou.org/english/index.htm), classification is shown: ly, lymph node invasion; venous invasion; pStage, pathological stage. ^*^Patients determined to be positive.

## DISCUSSION

Exhaled volatile organic compounds (VOCs) have been reported as biomarkers of certain gastrointestinal disorders, such as inflammatory bowel disease, ulcerative colitis, and necrotizing enterocolitis [19–22]. Studies on VOCs from patients with cancer have identified several substances in the exhaled breath of patients with lung and breast cancer using gas chromatography/mass spectroscopy [23–30]. Although these data are promising, such biomarkers are not commonly used in clinical practice, mainly because of the technical difficulties in measuring exhaled substances. Sniffer dogs have been employed to measure such exhaled substances from patients with cancer [8, 9, 31]. With the observation that sniffer dogs could distinguish patients with cancer through the scent of not only their stools but also exhaled breath, they speculated that cancer-specific substances may be circulating throughout the body of patients with cancer. The present study showed that the cancer detection system involving *C. elegans* can be an alternative method for measuring cancer-specific substances. This biological diagnostic system seemed to be economical, painless, rapid, and convenient. Therefore, the clinical application of *C. elegans* may be easier compart to that with sniffer dogs. Considering previous studies on other cancers, several substances may be involved in the scent test using *C. elegans*, including volatile n-alkanes, such as pentane, hexane, and longer-chain alkanes, derived from carcinoma-associated fibroblasts in breast cancer cases [24]. Moreover, given that the Ras-MAPK pathway regulates meiosis and spout formation in *C. elegans*, such a pathway has been shown to reduce chemotaxis to odorants, and the exposure of the odorant isoamyl alcohol to *C. elegans* resulted in the activation of the MAP kinase in olfactory neurons within 10 s, suggesting that the Ras-MAPK pathway may be a candidate pathway involved in scent detection using *C. elegans*, although the underlying mechanisms for chemotaxis remain to be elucidated [13, 14, 32, 33]. Eventually, recent study proposed that 2-octonone and pentanal levels were increased in urine of prostate cancer patients, compared to healthy controls, suggesting that both 2-octonone and pentanal higher levels in urine may be biomarkers of prostate cancer [34]. Moreover, very recent study of pancreatic ductal adenocarcinoma indicated that possible VOCs and metabolite biomarkers in urine were acetone, 2-pantanone, 4-methyl-2-heptanone, D-limonene, and levomenthol, though both chronic pancreatitis and PDAC shared common VOCs, so in the previous study, the authors could not fully discriminate both chronic pancreatitis and PDAC using GC-TOF-MS [35]. It is suggested that *C. elegans* in N-NOSE could sense a more complex pattern of VOCs and metabolites in urines and showed a chemotaxis response; that *C. elegans* may be superior to the GC-TOF-MS with headspace analysis approach with urine samples. Furthermore, a study reported patterns of VOCs in urine of PDAC patients, but without identifying specific molecules [36]. The study showed different patterns of VOCs, which could be distinguished between pre-cancer with acute pancreatitis and advanced PDAC cases, suggesting the measurement of VOCs before and during pancreatic cancer stages may make clear different patterns to healthy controls [36].

Importantly, this study showed significant differences in the chemotaxis assays of preoperative samples between patients with early-stage PDAC and healthy volunteers but to difference between preoperative and postoperative samples. One possible explanation for this observation could be that patients with PDAC may possess a special constitution that would produce special metabolomes, which may produce a special scent in patients with cancer. This explanation could help us understand that cancer patients possess certain substances, even with a very small cancerous lesion. Another explanation could be the presence of substances that produce scents in patients with cancer and persist even 1 month after surgical resection. Given the lack of data regarding the nature or half-life of the substances, we cannot determine which hypothesis is correct.

Investigating patients with very-early-stage PDAC is important for identifying biomarkers for early-stage PDAC. Paradoxically, however, analyzing samples from such patients is difficult given the rarity of such cases. Melo et al. reported that glypican-1 could detect early pancreatic cancer in only four of the patients with stage I disease [37]. Therefore, we established a nationwide clinical group that involved high-volume centers throughout Japan and prospectively obtained urine samples from patients with very-early-stage PDAC (stage 0 or IA). Particularly, special attention was provided to ensure the quality of the samples in order to obtain uniform and sufficient quality.

This study has several limitations worth noting. First, the number of patients enrolled remains insufficient, despite having established a clinical group to effectively obtain samples. Thus, the evaluation of factors (e.g., age, sex, and other questionnaire items such as inflammatory disorders) between patients and volunteers was limited. In the future, another validation study should be attempted to clarify the utility of this method in clinical practice. Second, it remained unclear whether the scent of the cancer patients was indeed derived from the cancerous lesions or from the metabolic disorders caused by the impaired pancreatic function. Nevertheless, chemotaxis assays have been used in other types of cancers, including colorectal, stomach, and breast cancers, implying that impaired pancreatic function could not have been the reason for the scent of patients with PDAC. Third, the cutoff value of this cancer detection system for early PDAC has yet to be determined. To utilize this system as a screening test for patients with early-stage PDAC, higher specificity is imperative. Despite the high specificity in previous studies [15, 17], an excessive number of participants would need to be sent for detailed examination for PDAC. Moreover, the suitable urine dilution for early PDAC remains under investigation.

In conclusion, the current study observed higher chemotaxis of *C. elegans* in patients with very-early-stage PDAC, suggesting its potential for use as a standard method for detecting early-stage cancer. Nonetheless, the underlying mechanisms for this chemotaxis should be clarified in order to obtain information that could help elucidate the biological characteristics of cancer.

## MATERIALS AND METHODS

### Patients and healthy volunteers

To effectively recruit the exceedingly rare patients with early-stage PDAC (stage 0 or IA), we organized a nationwide group that comprised seven high-volume centers. We recruited stage IA cases based on the 7th edition of the Union for International Cancer Control (UICC), which is more stringent for early-stage cancer. Patients with PDAC were enrolled from October 2015 to June 2019. No restrictions were placed on meals or activities for sampling. The participants were required to be > 20 years old and were asked to complete a questionnaire on factors that could influence the volatile molecules in their urine or serum samples. These factors included age; physical symptoms, such as appetite, weariness, headache, chest, or abdominal distention, cough, bloody feces, constipation, and diarrhea; pregnancy; history of cancer treatment; current use of medicine; alcohol consumption of three or more days per week; and smoking within the previous 2 weeks. The exclusion criteria included participants who had undergone cancer surgery within the previous year, those who were not examined for cancer recurrence despite having undergone cancer surgery more than 5 years previously, and those currently receiving chemotherapy. Given our suspicion that chemotherapy or operation would change urine chemicals in patients with cancer, we sought patients who had not yet undergone any treatment. A serial number was written on each sample tube at the time of collection to identify individual information.

### Blood serum and urine sampling

Special attention was provided to maintain the quality of the serum and urine samples, which were harvested early in the morning at each hospital from patients with stage 0 or IA PDAC. The samples were immediately placed on ice and sent by jet air or limited super express train to Osaka University. Each serum sample was separated from whole blood. The separated serum was placed in a 1-mL polypropylene screw cap tube and stored at −80°C until use. Urine samples were processed and stored in the same manner.

### Tumor marker determination

Tumor marker concentrations were determined at SRL Inc. Serum CA19-9 and CEA concentrations were determined using chemiluminescent enzyme immunoassay. The cutoff values for serum CA19-9 and CEA were 37 IU/mL and 5.0 ng/mL.

### Worm cultures and strains

*C. elegans* strains were cultured at 20°C under standard conditions on NGM plates with *Escherichia coli* NA22, which grows in thick layers, serves as a suitable food source for large-scale worm cultures, and have been used for chemotaxis analyses [32, 33, 38, 39]. The strains used in this study were wild-type N2.

### *C.* elegans cancer detection test

A blinded test was conducted with the assayers not knowing the origin of the urine samples. In the chemotaxis assays, 50–100 approximately synchronized young adults participated, after which the chemotaxis index was calculated as previously described [13–15, 33, 38, 40]. We placed 1 μL of urine on two spots on one end of the assay plates (2% agar, 5 mM KPO, 1 mM CaCl, and 1 mM MgSO) and added 0.5 μL of 1-M sodium azide on two spots on both ends of the plates. Animals were collected, washed three times with basal buffer (0.05% gelatin, 5 mM KPO_4_, 1 mM CaCl_2_, and 1 mM MgSO_4_), and spotted to the center of the plates. After 30 min, the number of nematodes was counted, and the chemotaxis index was calculated as follows:

Chemotaxis index=(A−B)/(A+B),

where A is the number of nematodes on the urine-spotted side of the plate and B is the number of nematodes on the opposite side. The average chemotaxis indices of more than 10 assay plates were determined. Maintaining the room temperature at 23°C ± 1°C was important. The urine samples stored at −80°C were thawed and kept at room temperature immediately before the assays.

The engaging behavior of *C. elegans* toward favorite smells would suggest a positive chemotaxis index. Furthermore, the chemotaxis index is correlated with the degree of odor concentrations, and positive peaks appear in accordance with odor concentrations when an attractive odorant is present in the samples. A previous study [15] analyzed 10-fold diluted urine to more thoroughly investigate the olfactory responses of *C. elegans*. In the current study, multiple urine concentrations (10-fold and 100-fold) were used to analyzed for the presence of positive peaks in the average chemotaxis indices.

### Ethics approval

This study was approved by the institutional review board of Osaka University Hospital. All patients and volunteers provided written informed consent.

### Statistical analysis

Differences in characteristics, laboratory data, and tumor markers between control participants and those with cancer were examined using the Wilcoxon signed-rank test (if a pair exists) and Wilcoxon rank-sum test (if the pair does not exist) for dichotomized variables. A *p* value of < 0.05 was considered statistically significant. Cancer staging was based on the UICC criteria.

## SUPPLEMENTARY MATERIALS


