# Supplementary material for: Scent test using Caenorhabditis elegans to screen for early-stage pancreatic cancer
Source: Oncotarget. 2021 Aug 17;12(17):1687–96. doi: 10.18632/oncotarget.28035 (PMC8378769; doi:10.18632/oncotarget.28035)
Supplement: Supplementary file 1 [file oncotarget-12-1687-s001.pdf]

Scent test using *Caenorhabditis elegans* to screen for early-stage pancreatic cancer

SUPPLEMENTARY MATERIALS

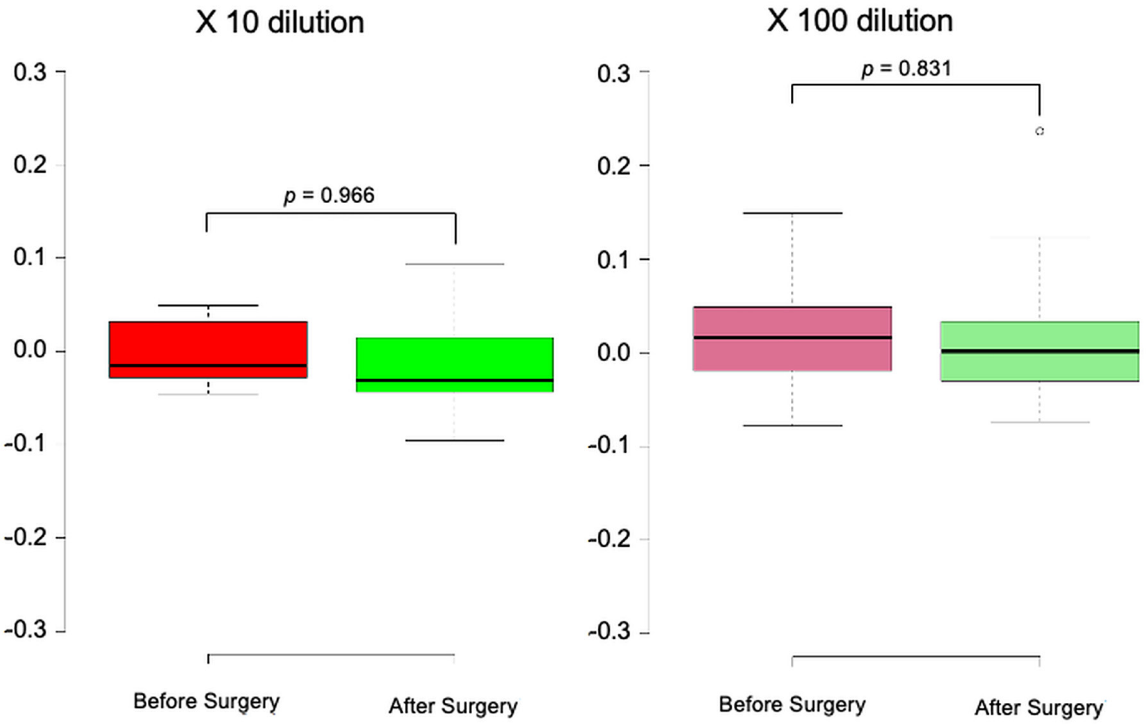

Supplementary Figure 1: Scent test using *Caenorhabditis elegans* for the diagnosis of early pancreatic ductal adenocarcinoma in the blinded study.
